# Risk of Insulin Resistance: Comparison of the Commerce vs. Industry Sector and Associated Variables

**DOI:** 10.3390/diseases13050150

**Published:** 2025-05-14

**Authors:** María Pilar Fernández-Figares Vicioso, Pere Riutord Sbert, Ángel Arturo López-González, José Ignacio Ramírez-Manent, José Luis del Barrio Fernández, María Teófila Vicente Herrero

**Affiliations:** 1 Obesity and Metabolic Syndrome Group, Spanish Association of Specialists in Occupational Medicine, 28012 Madrid, Spain; pfigares@gmail.com (M.P.F.-F.V.); correoteo@gmail.com (M.T.V.H.); 2 ADEMA-Health Group of IUNICS, University of Balearic Islands, 07122 Palma, Spain; pereriutord@gmail.com (P.R.S.); jignacioramirez@telefonica.net (J.I.R.-M.); 3 Health Research Institute of the Balearic Islands (IDISBA), 07120 Palma, Spain; 4 Faculty of Medicine, University of Balearic Islands, 07122 Palma, Spain; 5 Faculty of Health Sciences, Rey Juan Carlos University, 28032 Madrid, Spain; jose.delbarrio@urjc.es

**Keywords:** insulin resistance, sociodemographic variables, Mediterranean diet, physical activity, occupational health

## Abstract

Background: Insulin resistance (IR) is a key metabolic alteration that precedes type 2 diabetes and is closely linked to obesity and lifestyle factors. Occupational context may influence IR risk through variations in physical activity, diet, and socioeconomic determinants. Objective: To compare the risk of insulin resistance between workers in the commerce and industry sectors and identify associated sociodemographic and lifestyle factors, in order to improve their occupational health. Methods: This cross-sectional study analyzed data from 56,856 Spanish workers, assessing four IR-related indices: Triglyceride-Glucose Index (TyG), TyG-BMI (Triglyceride-Glucose Body Mass Index), Metabolic Score for Insulin Resistance (METS-IR), and the Single-Point Insulin Sensitivity Estimator (SPISE-IR). The analysis was stratified by sex and sector (commerce vs. industry) and included assessments of age, education level, physical activity, adherence to the Mediterranean diet, and smoking status. Multinomial logistic regressions were performed to determine the factors associated with high IR scores. Results: Across all IR indicators, industry workers—particularly men—presented higher mean values and greater prevalence of high-risk scores compared to those in commerce. Women showed lower values overall but also reflected sector-based differences. In both sexes, non-physical activity, non-adherence to the Mediterranean diet, and smoking were consistently associated with higher IR risk. Males exhibited significantly higher odds of elevated TyG (OR = 2.59, 95% CI: 2.41–2.78), while physical inactivity and poor diet emerged as the most powerful modifiable predictors across all scales (e.g., OR = 10.45 for TyG, OR = 12.33 for TyG-BMI). Industry sector was independently associated with higher odds of insulin resistance compared to commerce. Conclusions: Insulin resistance is more prevalent among industrial workers, especially men and those with unhealthy lifestyles. Occupational health strategies should target sector-specific risk profiles, emphasizing physical activity and dietary interventions.

## 1. Introduction

Insulin resistance (IR) is a complex pathophysiological phenomenon that has gained increasing importance in the fields of preventive medicine and biomedical research, due to its central role in the onset and progression of various non-communicable chronic diseases [1]. Among these, type 2 diabetes mellitus (T2DM) [2], non-alcoholic fatty liver disease (NAFLD) [3], dyslipidemia [4], arterial hypertension [5], and cardiovascular diseases [6] are particularly notable, as well as certain types of cancer [7]. It is estimated that a significant proportion of the global adult population—nearly one in four individuals—exhibits some degree of insulin resistance, often without overt clinical manifestations, which underscores its relevance as a major public health challenge [8].

Insulin, secreted by pancreatic β-cells, is essential for maintaining energy homeostasis [9]. It facilitates glucose uptake in insulin-sensitive tissues such as skeletal muscle and adipose tissue [10], regulates lipogenesis [11] and protein synthesis [12], and inhibits hepatic gluconeogenesis [13]. In the context of IR, these physiological functions become impaired, leading to compensatory hyperinsulinemia and systemic metabolic dysfunction [14]. IR is a multifactorial process influenced by genetic, epigenetic, environmental, and behavioral factors [15,16,17,18]. Excess visceral fat plays a central role by disrupting insulin signaling through the release of free fatty acids and pro-inflammatory molecules [19,20,21], such as TNF-α and IL-6, along with a reduction in protective adipokines like adiponectin [22]. At the molecular level, IR is associated with alterations in the insulin receptor signaling cascade [23,24,25], ultimately impairing cellular glucose uptake [26].

IR is influenced by multiple factors, with central obesity being one of the most relevant [27]. Other contributing factors include physical inactivity [28], a high-calorie diet [29], chronic stress [30], aging [31], sleep disorders [32], and genetic predisposition [33]. Additionally, certain ethnic groups, such as Native Americans, Afro-descendants, and people from Southeast Asia, are at higher risk due to genetic variants that affect insulin action [34].

The socioeconomic environment also influences the risk of IR. Factors such as poverty, food insecurity, disorganized urbanization, and limited access to healthy foods promote obesogenic environments [35]. Furthermore, chronic stress, precarious working conditions, and adverse childhood experiences increase vulnerability to metabolic disorders related to insulin resistance [36].

Despite its high prevalence, IR often remains underdiagnosed, partly due to the absence of universal diagnostic criteria. The most accurate method for assessing IR is the hyperinsulinemic-euglycemic clamp, considered the gold standard, although its complexity restricts its use to research settings [37]. In routine clinical practice, indirect methods are more commonly used, such as HOMA-IR [38], which relies on fasting glucose and insulin levels, as well as QUICKI [39] and the Matsuda index [40], derived from oral glucose tolerance tests.

In recent years, novel indices that do not require insulin measurements have emerged and have been validated as reliable markers of IR in population-based studies:

- TyG index (Triglyceride-Glucose index) [41]: This marker has proven to be a practical and cost-effective substitute for detecting insulin resistance, with good correlation to the hyperinsulinemic clamp.
- METS-IR (Metabolic Score for Insulin Resistance) [42]: This index incorporates BMI, glucose, triglycerides, and HDL cholesterol into its formula and has shown high predictive capacity for identifying IR across diverse populations.
- SPISE (Single Point Insulin Sensitivity Estimator) [43]: Designed primarily for adolescents and young adults, it is based on triglycerides, HDL cholesterol, and body mass index. It has demonstrated utility in estimating insulin sensitivity without requiring insulin measurements, making it especially useful for large cohorts or low-resource settings.

Additionally, several clinical and biochemical markers have been associated with IR, including waist circumference, the triglyceride/HDL ratio, high-sensitivity C-reactive protein (hs-CRP), and proteins such as fetuin-A, resistin, and RBP4 [44,45,46,47].

IR contributes not only to the development of type 2 diabetes but also to significant metabolic and systemic dysfunctions. It is associated with endothelial dysfunction [48], inflammation [49], and increased platelet reactivity [50], all of which elevate cardiovascular risk. IR is linked to progressive forms of non-alcoholic fatty liver disease (NAFLD), such as non-alcoholic steatohepatitis (NASH) [51], as well as to reproductive disorders including polycystic ovary syndrome (PCOS) [52], male infertility [53], gestational diabetes [54], and preeclampsia [55]. Recent evidence also associates IR with cognitive decline and neurodegenerative diseases, such as Alzheimer’s disease [56].

The rising prevalence of IR is placing an increasing burden on healthcare systems. Early detection and preventive strategies are key to mitigating its impact. Numerous studies have shown that lifestyle modifications—including weight loss, physical activity, a healthy diet, and stress management—can reverse IR in its early stages [57].

IR is a central pathophysiological factor in multiple chronic diseases. Addressing it requires a multidisciplinary approach that considers both biological mechanisms and social determinants of health. In a context marked by sedentary lifestyles and social inequality, effective and sustainable preventive policies are essential. Only coordinated action across research, clinical practice, and public health can reduce the silent yet profoundly disruptive impact of this metabolic alteration.

This study aimed to explore differences in the risk of IR between workers in the commerce and industry sectors by analyzing a broad range of sociodemographic, clinical, and behavioral variables. Using validated surrogate markers—TyG, TyG-BMI (Triglyceride-Glucose Body Mass Index), METS-IR, and SPISE—the findings provide a comprehensive overview of how occupational environments and lifestyle factors interact in shaping metabolic risk. In order to improve the occupational health of these workers.

## 2. Methods

### 2.1. Study Design and Population

**Figure 1:**
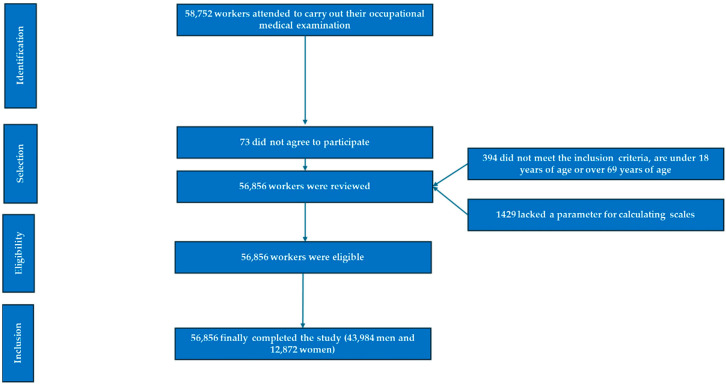
PRISMA diagram illustrating the participant selection process for this study.

A cross-sectional descriptive study was conducted, involving a cohort of 56,856 working individuals from the commerce and industry sectors. Of the total participants, 43,984 were men and 12,872 were women. The sample comprised individuals who attended occupational health examinations between January 2017 and December 2019. The selection process for study participants is illustrated in the corresponding flowchart (Figure 1).

### 2.2. Eligibility Criteria

To be included in the study, individuals had to meet the following criteria:

- Be between 18 and 69 years of age.
- Provide informed consent for participation.
- Explicitly authorize the use of their data for research purposes.
- Be employed by companies included in the study and not be on medical leave at the time of assessment.

### 2.3. Data Collection

Information was collected by trained healthcare professionals affiliated with the occupational health services of the participating companies. Data collection was carried out through three main strategies:

1. Structured Clinical Interview: Sociodemographic variables (age, sex, education level) and health-related behaviors such as smoking, dietary patterns, and physical activity were recorded.
2. Physical and Clinical Measurements: Anthropometric data (weight, height, waist and hip circumferences) and blood pressure parameters (systolic and diastolic) were collected.
3. Biochemical Analyses: Blood lipid profiles and glucose levels were assessed.

To ensure data quality and reliability, all measurements were standardized according to established technical protocols:

- Weight and height: Measured with the subject barefoot and wearing only underwear, standing upright, using a SECA 700 scale (SECA, Chino, CA, USA) and a SECA 220 stadiometer (SECA, Chino, CA, USA).
- Body circumferences: Measured using SECA measuring tape (SECA, Chino, CA, USA). Waist circumference was measured at the level of the last floating rib, while hip circumference was taken at the widest part of the buttocks. Both measurements were performed with the subject standing and abdomen relaxed.
- Blood pressure: Measured with an automatic sphygmomanometer OMRON-M3 (OMRON, Osaka, Japan), with the participant seated and after a minimum of ten minutes of rest. Three consecutive readings were taken at one-minute intervals, and the average of the three was recorded.
- Blood samples: Collected via venipuncture after a minimum 12-h fast. The samples were processed as follows: An 8.5 mL BD SST II Vacutainer serum tube with gel separator (reference BD 366468) was used. The samples were transported to the laboratory in a refrigerated container (between 5 and 10 degrees Celsius). Upon arrival, the samples were centrifuged within two hours of collection and immediately analyzed using an automated analyzer [58,59]. LDL was calculated using the Friedewald formula, except in cases with triglycerides ≥400 mg/dL, for which direct measurement was used [60]. All biochemical variables are reported in milligrams per deciliter (mg/dL).

#### 2.3.1. Operational Definitions of Variables

- Biological sex: Classified as male or female.
- Education level: Grouped into two categories: basic education (primary) and higher education (secondary or tertiary).
- Tobacco use: Individuals were considered smokers if they had smoked daily in the past 30 days or had quit smoking within the last 12 months.
- Adherence to the Mediterranean diet: Assessed using a 14-item binary questionnaire (score 0–1). A score of 9 or higher indicated good adherence [61].
- Physical activity: Measured using the International Physical Activity Questionnaire (IPAQ), which evaluates the frequency, duration, and intensity of activities performed during the previous seven days [62].

#### 2.3.2. Insulin Resistance Risk Scales

The Triglyceride-Glucose Index (TyG) is derived using the natural logarithm of the product of fasting triglyceride in mg/dL and glucose in mg/dL levels divided by two: TyG = ln[(triglycerides × glucose)/2]. A threshold value of 8.5 or greater is commonly interpreted as indicative of elevated insulin resistance risk [63].

The Single-Point Insulin Sensitivity Estimator (SPISE) is computed with the following formula: SPISE = (600 × HDL^0.185^)/(triglycerides^0.2^ × BMI^1.338^). Insulin resistance according to this model is expressed as SPISE-IR = 10/SPISE, and individuals scoring 1.51 or higher on this inverse scale are classified as high-risk [64].

The Metabolic Score for Insulin Resistance (METS-IR) incorporates lipid and glycemic variables alongside body mass index. It is calculated using the formula: METS-IR = ln(2 × glucose) + (triglycerides × BMI)/ln(HDL cholesterol). A value of 50 or above suggests an increased likelihood of insulin resistance [65].

### 2.4. Statistical Analysis

Univariate analysis of categorical variables was performed using absolute frequencies and percentages. Continuous variables were summarized using means and standard deviations due to their normal distribution. Group comparisons were conducted using the chi-square test or Fisher’s exact test when appropriate. The Student’s t-test was used for mean comparisons. Multivariable analysis was conducted using multinomial logistic regression to explore associations between independent variables and categories of insulin resistance risk scales, calculating odds ratios with 95% confidence intervals. Model fit was assessed using the Hosmer–Lemeshow test. Stratified analyses were conducted to identify potential confounding factors. The model was adjusted for age, sex, education, diet, physical activity, and smoking; however, no significant effects were detected. All statistical analyses were conducted using SPSS software, version 29.0 for Windows (IBM Corp., New York, NY, USA), with a significance level set at *p* < 0.05.

## 3. Results

Significant differences were observed between commerce and industry workers, particularly among women. Female industry workers were older, heavier, and had higher waist circumference, blood pressure, and a less favorable lipid profile compared to those in commerce. In men, differences were generally smaller, though industry workers showed slightly higher systolic blood pressure, glucose, and lipid levels.

Younger age and higher physical activity and Mediterranean diet adherence were more common in the commerce sector across both sexes. Educational attainment was lower among commerce workers, especially women. Smoking was more prevalent among industrial men, while no difference was found in women (Table 1).

These findings underscore notable sector-specific differences in metabolic and lifestyle profiles, especially among women, with potential implications for occupational health strategies.

The analysis of insulin resistance (IR) indices across sectors revealed consistent differences by sex, age, education level, and health behaviors. Across all age groups and scales (TyG, TyG-BMI, METS-IR, SPISE), both men and women in the industry sector showed higher mean values and a greater prevalence of elevated IR, indicating a less favorable metabolic profile compared to commerce workers.

In men, IR increased progressively with age across all scales, with the highest means and proportions of high values observed in those aged 60–69. Women followed a similar age-related trend, though the differences were more pronounced in industry workers, particularly for the TyG-BMI and SPISE-IR indices.

Lower educational attainment was associated with higher IR, especially among women. Those with only elementary education consistently showed higher means and prevalence rates across all indices, more markedly so in the industrial sector.

Physical activity (PhA) and adherence to the Mediterranean diet (MD) were strongly associated with lower IR levels. Physically active individuals and those adhering to MD consistently showed significantly lower mean scores and lower prevalence of elevated IR across all scales and both sectors, with more favorable profiles in commerce.

Smoking was associated with higher IR in both sexes. Smokers in the industry sector showed particularly elevated mean values and higher proportions of IR, especially for TyG-BMI and SPISE-IR, suggesting a potential interaction between occupational environment and unhealthy habits.

These findings underscore the influence of occupational sector, lifestyle behaviors, and sociodemographic variables on insulin resistance, with consistently less favorable metabolic profiles in industry workers—particularly among older, less educated, sedentary, and smoking individuals. These insights may inform targeted prevention strategies and workplace health interventions. (Table 2 and Table 3).

The multinomial logistic regression analysis identified several sociodemographic and lifestyle factors independently associated with elevated insulin resistance (IR) as measured by TyG, TyG-BMI, METS-IR, and SPISE-IR indices (Table 4).

Sex differences were prominent: men had significantly higher odds of elevated TyG (OR: 2.59; 95% CI: 2.41–2.78) and TyG-BMI, whereas they were less likely to present high METS-IR values (OR: 0.84; 95% CI: 0.77–0.92). The association with SPISE-IR was modest but positive (OR: 1.11).

Age showed a clear dose-response pattern. Compared to the youngest group (18–29 years), older individuals exhibited progressively higher odds of elevated IR across all indices. Notably, those aged 60–69 had the highest odds, especially for METS-IR (OR: 2.37; 95% CI: 1.95–2.79) and SPISE-IR (OR: 1.96; 95% CI: 1.66–2.27), highlighting age as a strong determinant of metabolic risk.

Educational level was modestly associated with IR. Individuals with high school education showed slightly higher odds across all indices, suggesting that education alone may not fully mitigate metabolic risk.

Occupational sector was an independent predictor: workers in the industry sector had significantly higher odds of elevated IR for all indices, particularly for METS-IR (OR: 1.37; 95% CI: 1.28–1.47), suggesting sector-specific environmental or behavioral risk exposures. The differences between the industrial and commercial sectors in men were statistically significant, although less pronounced than in women. This suggests that, in males, the influence of the occupational sector on the analyzed indicators may be less determinant.

Lifestyle behaviors showed the strongest associations. Lack of physical activity was the most influential factor, with ORs ranging from 8.31 (SPISE-IR) to 12.33 (TyG-BMI), indicating a ten- to twelvefold increased risk of insulin resistance among inactive individuals. Similarly, low adherence to a Mediterranean diet was strongly associated with elevated IR (e.g., OR for TyG-BMI: 5.29; 95% CI: 4.80–5.79).

Finally, smoking was independently associated with higher odds of elevated IR, although the effect sizes were smaller (e.g., OR for TyG: 1.53; 95% CI: 1.46–1.61). The effect of the occupational sector was independent of diet, education, physical activity, and smoking, as shown by the multivariable regression analysis.

These findings emphasize the critical role of modifiable behaviors—especially physical inactivity and poor dietary habits—in shaping insulin resistance risk, independently of sex, age, education, and occupational context. Targeted interventions at the workplace and population levels may help address these disparities.

## 4. Discussion

In all IR indices, a consistent pattern was identified: workers in the industrial sector, both men and women, exhibited significantly higher mean values and prevalence rates than their counterparts in the commerce sector. These differences were consistent across all age groups, educational levels, and lifestyle categories.

Multinomial logistic regression analyses, adjusted for potential confounders (age, sex, physical activity, diet, and smoking), confirmed this association. Employment in the industrial sector increased the likelihood of elevated values in TyG (OR = 1.23), TyG-BMI (OR = 1.17), METS-IR (OR = 1.37), and SPISE-IR (OR = 1.20).

The higher risk observed in the industrial sector may be partly explained by the inherent characteristics of the work environment. Industrial occupations often involve physically demanding and repetitive tasks, exposure to toxic agents, adverse working conditions, and irregular or night shifts. These factors can disrupt circadian rhythms and hormonal balance, promote metabolic dysfunction, and contribute to the development of IR, even in the absence of classical risk factors such as poor diet or physical inactivity. Furthermore, low job autonomy and psychosocial stress—common in such environments—may exacerbate metabolic dysfunction through activation of the hypothalamic-pituitary-adrenal axis and systemic inflammation [66,67].

Across all indices used, men exhibited significantly higher IR values and prevalence than women. For instance, the likelihood of elevated TyG was more than twice as high in men compared to women (OR = 2.59), even after adjustment for covariates. While both sexes were affected by sectoral differences, the gap between commerce and industry was particularly pronounced among older women and those with lower educational attainment. These findings align with existing evidence on sexual dimorphism in insulin sensitivity [68], adipose tissue distribution [69], and hormonal regulation [70]. Premenopausal women are partly protected due to the estrogenic effects on glucose metabolism and fat distribution [71], but this advantage tends to decline with age. Moreover, the industrial work environment appears to blunt part of this protective effect, as reflected in the higher SPISE-IR values observed among industrial-sector women.

Age emerged as one of the strongest predictors of IR across all scales analyzed. Participants aged 60–69 were nearly twice as likely to show elevated values in TyG and SPISE-IR compared to the 18–29 age group, and 2.4 times more likely in the case of METS-IR. This progressive increase reflects the natural decline in insulin sensitivity associated with aging, likely exacerbated by the cumulative burden of adverse metabolic factors over time [72].

A noteworthy interaction was found between age and sector: older individuals in the industrial sector had the highest mean values and prevalence across all four indices, highlighting the urgent need for targeted preventive strategies for older workers, who may bear a double burden of risk—due to both aging and work conditions.

Participants with higher educational attainment consistently exhibited lower values and prevalence of insulin resistance. For example, those with secondary education had a 10–15% lower likelihood of presenting abnormal values in the indices compared to individuals with only primary education. These results are consistent with the well-established inverse relationship between education level and cardiometabolic risk, likely mediated by higher health literacy, healthier behaviors, and greater access to preventive care [73].

Notably, the protective effect of education was more evident in the commerce sector, possibly due to the combination of more sedentary work and healthier dietary and behavioral patterns among more educated employees. In contrast, this effect was attenuated in the industrial sector, perhaps due to the intensity of physical and environmental stressors in that occupational setting.

Although our study considered educational level as a relevant sociodemographic variable, it would have been highly valuable to also include data on individual or household income. This information could provide a more comprehensive understanding of the influence of socioeconomic status on IR values, as previous research suggests an association between lower income and higher metabolic risk. However, due to the absence of this variable in our sample, we were unable to assess it. We recommend that future studies incorporate income-related variables to allow for a more comprehensive analysis. Regarding access to healthcare services, it is important to note that Spain has a public and universal National Health System, which provides free healthcare to the entire population regardless of economic status. Nevertheless, educational level may influence the effective use of these services, as individuals with lower educational attainment are generally less engaged in preventive healthcare and health promotion activities.

The strongest protective effects against IR were observed among workers who engaged in regular physical activity and adhered closely to the Mediterranean diet. These two healthy behaviors were significantly associated with lower mean values and prevalence of alterations across all IR indices. Lack of physical activity was one of the most potent predictors of insulin resistance: those not engaging in regular exercise were between 8 and 12 times more likely to show abnormal values, depending on the scale analyzed. For instance, inactive workers had an OR of 12.33 for elevated TyG-BMI and 11.87 for high METS-IR, confirming the central role of sedentary behavior as a modifiable risk factor—findings supported by recent studies in this field [74].

Similarly, low adherence to the Mediterranean diet increased the likelihood of presenting IR by 3.6 times (SPISE) to more than 5 times (TyG-BMI and METS-IR). These findings, corroborated by several studies [75], underscore the importance of diet quality for metabolic health, particularly in work environments where access to healthy food may be constrained by time or economic factors.

Current smokers exhibited higher values in all IR indices, with significantly increased odds of metabolic alterations. Although the effect size was smaller than that of diet or physical activity, the association remained statistically significant. This aligns with previous research showing that smoking disrupts insulin signaling, increases oxidative stress, and promotes abdominal fat accumulation [76,77].

Interestingly, the magnitude of smoking’s effect varied by sex and sector. Among male smokers in the industrial sector, the prevalence of elevated METS-IR reached 10%, nearly double that of non-smokers in the same setting. This suggests a possible interaction between smoking and work-related stress factors, in which adverse occupational conditions may induce a state of chronic anxiety that promotes the maintenance or increase of smoking habits, which in turn could synergistically worsen the observed metabolic outcomes [78]. In this regard, it would be interesting for future studies to include the assessment of molecular markers such as cortisol or high-sensitivity C-reactive protein.

This study employed four validated non-invasive indices as indirect markers of insulin resistance: TyG, TyG-BMI, METS-IR, and SPISE. Each of these indices captures specific metabolic dimensions, enabling a more nuanced assessment of insulin resistance risk across different population subgroups. The consistency of the findings across all indices strengthens the validity of the results and supports their potential application in occupational health surveillance programs. This multidimensional approach enhances early identification of individuals at metabolic risk, facilitating targeted interventions in workplace settings.

- TyG and TyG-BMI were highly sensitive to adiposity and dyslipidemia, proving especially useful in identifying risk among overweight individuals and those with low physical activity.
- METS-IR provided a more comprehensive perspective by incorporating HDL cholesterol.
- SPISE, though less commonly used, yielded relevant information on insulin sensitivity in younger and leaner individuals.

The findings of this study have significant implications for public health and occupational medicine. The higher metabolic risk observed in the industrial sector underscores the need for targeted interventions to improve workplace conditions, such as adjusting shift schedules, facilitating access to healthy food, and promoting physical activity.

Our results suggest that there may be differences in lifestyle habits between workers in the industrial and commercial sectors, which could influence their predisposition to developing insulin resistance. These differences may be related to factors such as work schedules, the level of physical or mental demands, ergonomic conditions, access to healthy food during the workday, or available time for physical activity. It would be highly valuable to extend this analysis to other professional sectors, such as education or healthcare, in order to better understand how the specific characteristics of each occupational setting may affect health-related behaviors. This information would support the design and implementation of targeted preventive interventions tailored to each sector, with the aim of improving metabolic health, overall well-being, and quality of life among workers, within more comprehensive and personalized occupational health strategies.

It is essential to strengthen health education initiatives and early detection programs, particularly targeting older workers and those with lower educational levels. Employers should promote workplace wellness programs that include structured physical activity, smoking cessation support, and nutritional counseling. Implementing workplace nutrition policies is key to improving dietary habits by offering healthy options in cafeterias and removing vending machines with ultra-processed products. Work schedules should also allow sufficient time for proper meals, avoiding rushed or unbalanced eating due to lack of breaks. Providing on-site fitness areas would encourage regular physical activity, benefiting cardiovascular health. Finally, incorporating stress management programs—such as mindfulness, yoga, or relaxation techniques—would help enhance employees’ emotional well-being. These integrated interventions can have a positive impact on workers’ overall health and quality of life.

From a public policy perspective, these findings support incorporating IR risk assessment into occupational health protocols, particularly in high-risk occupational sectors. The use of simple, validated indices such as TyG or METS-IR would enable early detection and timely intervention, helping to reduce the burden of chronic metabolic diseases.

### 4.1. Strengths of the Study

- Large sample size and sectoral representativeness: The study is based on a sample of over 56,000 workers from the commerce and industrial sectors, providing robust statistical power and allowing for reliable comparisons between groups. This broad scope facilitates the identification of genuine differences in IR risk and enhances the generalizability of findings within the labor context.
- Equitable inclusion of both sexes and a wide age range: The sample includes both men and women aged 18 to 69, enabling analysis of sex- and age-related differences and tracking the evolution of metabolic risk throughout the working life cycle.
- Simultaneous use of multiple validated IR indices: The combined use of TyG, TyG-BMI, METS-IR, and SPISE provides a more comprehensive evaluation of metabolic risk. Each index captures different IR-related dimensions (dyslipidemia, adiposity, insulin sensitivity), increasing the validity of findings and minimizing bias associated with reliance on a single marker.
- Comparative approach by economic sector: The sector-specific analysis (commerce vs. industry) represents a novel contribution. Few studies have explored how occupational type and structural characteristics (shifts, physical effort, stress, environment) relate to IR, lending added value to this research in the field of occupational health.
- Rigorous statistical control of confounding factors: The use of adjusted multinomial logistic regression models allows for assessment of independent associations while controlling for age, sex, education, physical activity, diet, and smoking—strengthening the reliability of the observed associations.
- Detailed assessment of lifestyle variables: The study incorporates key health behaviors (Mediterranean diet adherence, physical activity, smoking), often underrepresented in occupational health research, enabling the identification of meaningful associations between lifestyle and metabolic risk in workplace settings.
- Practical applicability to public health and occupational medicine: The indices employed are simple, cost-effective, and non-invasive, making the results easily translatable to screening, monitoring, and prevention programs within companies or organizations. This enhances the translational value of the study and its potential for large-scale interventions.

### 4.2. Study Limitations

- Cross-sectional design of the study prevents inferring causal relationships between the variables analyzed and IR rates. It would be interesting to conduct longitudinal studies to examine how IR evolves in these groups over time.
- Indirect measurement of IR: Although validated and widely used indices such as TyG, METS-IR, and SPISE were employed, they are indirect proxies and do not replace gold-standard methods like the hyperinsulinemic-euglycemic clamp.
- Self-reported data: Key variables such as physical activity, dietary adherence, and smoking status were self-reported, potentially introducing recall or social desirability bias.
- The pre- or postmenopausal status of the women was not recorded, which may influence glucose metabolism, body fat distribution, and insulin resistance.
- Lack of control for other occupational variables: Factors such as shift type, physical workload, occupational stress, or sleep quality were not included, despite their potential influence on metabolism and IR risk modulation.
- Limited generalizability: While the sample size is large, findings are limited to two occupational sectors and may not be generalizable to the entire working population or other socioeconomic settings.

## 5. Conclusions

The results of this study reveal significant differences in IR risk between workers in the commerce and industrial sectors. Industrial workers, regardless of sex, age, or education level, display less favorable metabolic profiles, with higher values and prevalence of alterations in TyG, TyG-BMI, METS-IR, and SPISE. These differences persist even after adjusting for sociodemographic factors and lifestyle, suggesting an independent influence of the work environment on metabolic risk.

Physical inactivity, low adherence to the Mediterranean diet, and smoking emerge as strongly modifiable factors associated with increased IR risk, with consistent odds ratios across all multivariate models. In this context, the scales used not only enable robust risk assessment but also serve as practical tools for epidemiological monitoring and decision-making in occupational health.

Overall, these findings support the need for tailored interventions aimed at promoting healthy lifestyles in work environments—particularly in the industrial sector—and reinforce the value of incorporating indices such as TyG, METS-IR, and SPISE in screening and prevention programs targeting cardiometabolic risk in working populations.

## Figures and Tables

**Table 1:** Characteristics of the population.

Header	Men			Women		
	Commerce n = 18,160	Industry n = 25,824		Commerce n = 9288	Industry n = 3584	
	Mean (SD)	Mean (SD)	*p*-Value	Mean (SD)	Mean (SD)	*p*-Value
Age (years)	39.5 (9.8)	39.4 (10.5)	0.225	35.9 (10.1)	41.6 (10.5)	<0.001
Height (cm)	175.0 (6.7)	173.9 (7.0)	<0.001	162.0 (6.4)	160.9 (6.5)	<0.001
Weight (kg)	81.5 (12.5)	81.3 (14.2)	0.064	65.3 (13.4)	68.8 (14.0)	<0.001
Waist circumference (cm)	87.5 (8.8)	87.7 (9.0)	0.121	73.7 (7.5)	75.1 (8.0)	<0.001
Hip circumference (cm)	100.6 (7.9)	99.6 (8.4)	<0.001	97.0 (8.9)	98.1 (9.4)	<0.001
SBP (mmHg)	122.6 (14.4)	124.5 (5.0)	0.024	112.6 (14.2)	117.9 (16.2)	<0.001
DBP (mmHg)	74.5 (10.2)	75.6 (10.5)	0.170	68.9 (9.8)	71.5 (10.7)	<0.001
Total cholesterol (mg/dL)	193.9 (37.4)	197.5 (38.6)	<0.001	189.4 (35.4)	201.1 (39.3)	<0.001
HDL-cholesterol (mg/dL)	51.1 (6.7)	51.4 (7.0)	<0.001	54.5 (7.9)	52.3 (7.5)	<0.001
LDL-cholesterol (mg/dL)	119.4 (37.7)	121.9 (37.2)	<0.001	117.7 (35.6)	130.6 (38.8)	<0.001
Triglycerides (mg/dL)	119.3 (81.3)	122.4 (84.6)	<0.001	85.4 (37.6)	90.8 (45.8)	<0.001
Glucose (mg/dL)	86.3 (11.9)	88.7 (12.9)	<0.001	84.2 (10.6)	84.3 (11.9)	0.210
	(%)	(%)	*p*-value	(%)	(%)	*p*-value
18–29 years	17.7	20.3	<0.001	32.1	16.5	<0.001
30–39 years	31.8	31.7		32.6	26.9	
40–49 years	33.6	28.5		23.6	31.0	
50–59 years	14.7	16.7		10.3	23.4	
60–69 years	2.2	2.8		1.4	2.2	
Elementary school	52.4	36.7	<0.001	90.1	83.7	<0.001
High school	47.6	63.3		9.9	16.3	
Non Physical activity	51.5	55.4	<0.001	42.7	59.4	<0.001
Yes Physical activity	48.5	44.6		57.7	40.6	
Non Mediterranean diet	56.1	59.8	<0.001	44.4	59.8	<0.001
Yes Mediterranean diet	43.9	40.2		55.6	40.2	
Non smokers	70.5	63.0	<0.001	68.0	67.2	0.181
Smokers	29.5	37.0		32.0	32.8	

SBP Systolic blood pressure. DBP Diastolic blood pressure. HDL High density lipoprotein. LDL Low density lipoprotein, SD Standard deviation.

**Table 2:** Mean of different scales of insulin resistance according sociodemographic variables and healthy habits by sex.

		TyG *				TyG-BMI *				METS-IR *				SPISE-IR *		
		Commerce		Industry		Commerce		Industry		Commerce		Industry		Commerce		Industry
Men	n	Mean (SD)	n	Mean (SD)	n	Mean (SD)	n	Mean (SD)	n	Mean (SD)	n	Mean (SD)	n	Mean (SD)	n	Mean (SD)
18–29 years	3224	8.1 (0.5)	5248	8.2 (0.5)	3224	204.2 (34.2)	5248	205.8 (39.3)	3224	34.7 (6.5)	5248	34.9 (5.8)	3224	1.4 (0.4)	5248	1.5 (0.4)
30–39 years	5768	8.3 (0.5)	8184	8.4 (0.6)	5768	217.5 (36.2)	8184	225.5 (43.8)	5768	37.3 (6.2)	8184	38.5 (7.5)	5768	1.6 (0.4)	8184	1.7 (0.5)
40–49 years	6104	8.5 (0.5)	7360	8.6 (0.6)	6104	231.0 (39.0)	7360	238.1 (45.2)	6104	39.6 (6.6)	7360	40.8 (7.7)	6104	1.7 (0.4)	7360	1.8 (0.5)
50–59 years	2664	8.6 (0.6)	4312	8.7 (0.5)	2664	241.0 (38.1)	4312	244.8 (38.7)	2664	41.2 (6.7)	4312	42.0 (6.7)	2664	1.8 (0.4)	4312	1.9 (0.4)
60–69 years	400	8.7 (0.5)	720	8.8 (0.5)	400	249.6 (35.4)	720	251.3 (34.4)	400	42.4 (6.0)	720	43.7 (6.7)	400	1.9 (0.5)	720	2.0 (0.5)
Elementary	9512	8.4 (0.6)	9480	8.5 (0.6)	9512	225.7 (37.8)	9480	229.0 (45.3)	9512	38.9 (6.6)	9480	39.1 (7.8)	9512	1.7 (0.4)	9480	1.8 (0.5)
High school	8648	8.3 (0.6)	16,344	8.4 (0.6)	8648	222.1 (40.3)	16,344	223.8 (42.0)	8648	38.0 (6.9)	16,344	38.6 (7.3)	8648	1.6 (0.4)	16,344	1.7 (0.5)
Non PhA	9344	8.6 (0.5)	14,304	8.7 (0.6)	9344	249.9 (35.1)	14,304	253.6 (39.6)	9344	42.9 (6.2)	14,304	43.5 (6.9)	9344	1.9 (0.4)	14,304	2.0 (0.4)
Yes PhA	8816	8.0 (0.4)	11,520	8.1 (0.4)	8816	194.3 (22.0)	11,520	196.2 (19.1)	8816	33.3 (3.6)	11,520	34.3 (3.2)	8816	1.3 (0.2)	11,520	1.4 (0.2)
Non MD	10,184	8.6 (0.6)	15,440	8.7 (0.6)	10,184	245.3 (37.2)	15,440	249.5 (41.2)	10,184	42.1 (6.6)	15,440	42.7 (7.2)	10,184	1.9 (0.4)	15,440	2.0 (0.5)
Yes MD	7976	8.0 (0.4)	10,384	8.1 (0.4)	7976	193.9 (22.0)	10,384	196.4 (19.5)	7976	33.3 (3.7)	10,384	33.8 (3.3)	7976	1.3 (0.2)	10,384	1.4 (0.2)
Non smokers	12,808	8.3 (0.6)	16,280	8.4 (0.6)	12,808	222.7 (43.2)	16,280	223.7 (41.8)	12,808	38.3 (7.7)	16,280	38.8 (7.8)	12,808	1.6 (0.4)	16,280	1.7 (0.5)
Smokers	5352	8.4 (0.6)	9544	8.5 (0.6)	5352	223.9 (38.0)	9544	229.7 (44.5)	5352	38.9 (7.8)	9544	39.3 (7.9)	5352	1.7 (0.5)	9544	1.8 (0.5)
Women	n	Mean (SD)	n	Mean (SD)	n	Mean (SD)	n	Mean (SD)	n	Mean (SD)	n	Mean (SD)	n	Mean (SD)	n	Mean (SD)
18–29 years	2984	7.9 (0.5)	592	8.0 (0.4)	2984	187.5 (40.2)	592	190.7 (40.1)	2984	32.0 (6.8)	592	33.1 (6.7)	2984	1.2 (0.4)	592	1.3 (0.4)
30–39 years	3024	8.0 (0.4)	960	8.1 (0.5)	3024	198.2 (42.7)	960	213.9 (50.1)	3024	34.0 (7.2)	960	36.7 (8.1)	3024	1.4 (0.4)	960	1.5 (0.5)
40–49 years	2192	8.1 (0.5)	1112	8.2 (0.5)	2192	210.9 (44.0)	1112	221.3 (49.8)	2192	36.4 (7.5)	1112	38.2 (8.2)	2192	1.5 (0.4)	1112	1.6 (0.5)
50–59 years	960	8.2 (0.5)	840	8.3 (0.5)	960	229.8 (59.5)	840	231.6 (42.2)	960	38.3 (5.8)	840	40.2 (7.2)	960	1.6 (0.7)	840	1.7 (0.4)
60–69 years	128	8.3 (0.5)	80	8.4 (0.5)	128	231.5 (33.8)	80	257.4 (63.9)	128	39.7 (10.1)	80	44.9 (10.8)	128	1.7 (0.6)	80	2.1 (0.7)
Elementary	8368	8.1 (0.5)	3000	8.2 (0.5)	8368	202.5 (44.4)	3000	219.6 (50.6)	8368	34.9 (6.8)	3000	37.9 (8.4)	8368	1.5 (0.5)	3000	1.6 (0.5)
High school	920	8.0 (0.4)	584	8.0 (0.5)	920	201.3 (38.9)	584	203.6 (41.3)	920	34.6 (8.0)	584	35.4 (7.1)	920	1.4 (0.4)	584	1.5 (0.4)
Non PhA	3928	8.3 (0.5)	2128	8.4 (0.5)	3928	237.6 (47.5)	2128	243.1 (46.6)	3928	40.8 (8.1)	2128	41.9 (7.7)	3928	1.8 (0.5)	2128	1.9 (0.5)
Yes PhA	5360	7.9 (0.4)	1456	8.0 (0.4)	5360	176.5 (19.8)	1456	178.8 (20.3)	5360	30.2 (3.4)	1456	30.9 (3.5)	5360	1.1 (0.2)	1456	1.2 (0.2)
Non MD	4120	8.2 (0.4)	2144	8.3 (0.5)	4120	232.8 (49.9)	2144	241.5 (47.5)	4120	39.9 (8.6)	2144	41.6 (7.9)	4120	1.7 (0.5)	2144	1.8 (0.5)
Yes MD	5168	7.8 (0.4)	1440	7.9 (0.4)	5168	178.0 (21.1)	1440	180.4 (22.7)	5168	30.5 (3.6)	1440	31.4 (3.9)	5168	1.1 (0.2)	1440	1.2 (0.2)
Non smokers	6320	8.0 (0.5)	2408	8.1 (0.5)	6320	199.1 (44.4)	2408	204.4 (43.6)	6320	34.0 (7.5)	2408	35.3 (7.3)	6320	1.4 (0.4)	2408	1.5 (0.5)
Smokers	2968	8.1 (0.5)	1176	8.2 (0.5)	2968	203.9 (46.3)	1176	223.1 (51.1)	2968	35.0 (8.0)	1176	38.5 (8.6)	2968	1.5 (0.5)	1176	1.6 (0.5)

TyG Triglyceride glucose index. BMI Body mass index. METS-IR Metabolic score for insulin resistance. SPISE-IR Single-point insulin sensitivity estimator. SD Standard deviation. SD Standard deviation. PhA Physical activity. (*) Statistical significance in all cases.

**Table 3:** Prevalence of high values of different scales of insulin resistance according sociodemographic variables and healthy habits by sex.

		TyG High *				TyG-BMI High *				METS-IR High *				SPISE-IR High *		
		Commerce		Industry		Commerce		Industry		Commerce		Industry		Commerce		Industry
Men	n	%	n	%	n	%	n	%	n	%	n	%	n	%	n	%
18–29 years	3224	9.4	5248	11.0	3224	6.9	5248	11.3	3224	1.2	5248	3.4	3224	3.0	5248	5.9
30–39 years	5768	16.1	8184	20.0	5768	14.0	8184	21.5	5768	4.3	8184	8.1	5768	6.4	8184	14.9
40–49 years	6104	27.5	7360	33.5	6104	23.1	7360	31.0	6104	6.3	7360	11.5	6104	13.6	7360	22.8
50–59 years	2664	36.0	4312	38.7	2664	35.4	4312	37.8	2664	11.1	4312	12.5	2664	20.1	4312	23.9
60–69 years	400	46.0	720	49.1	400	44.2	720	46.5	400	20.0	720	22.1	400	28.0	720	29.1
Elementary	9512	23.3	9480	23.6	9512	19.4	9480	20.1	9512	6.4	9480	9.2	9512	11.7	9480	14.6
High school	8648	21.3	16,344	24.9	8648	21.6	16,344	24.7	8648	5.2	16,344	7.8	8648	9.8	16,344	12.3
Non PhA	9344	42.3	14,304	42.7	9344	35.2	14,304	38.6	9344	10.0	14,304	13.8	9344	18.8	14,304	21.8
Yes PhA	8816	1.2	11,520	2.0	8816	4.1	11,520	4.3	8816	1.3	11,520	2.2	8816	2.2	11,520	4.3
Non MD	10,184	38.3	15,440	39.5	10,184	34.8	15,440	39.2	10,184	9.5	15,440	13.2	10,184	17.2	15,440	22.0
Yes MD	7976	1.9	10,384	2.4	7976	4.6	10,384	5.3	7976	2.2	10,384	3.3	7976	3.5	10,384	4.7
Non smokers	12,808	19.7	16280	22.8	12,808	19.6	16,280	24.7	12,808	5.0	16,280	6.6	12,808	9.8	16,280	13.9
Smokers	5352	27.6	9544	28.7	5352	20.2	9544	25.8	5352	7.6	9544	10.0	5352	12.9	9544	14.9
Women	n	%	n	%	n	%	n	%	n	%	n	%	n	%	n	%
18–29 years	2984	5.1	592	8.1	2984	7.0	592	9.5	2984	2.7	592	4.1	2984	4.0	592	4.2
30–39 years	3024	7.7	960	8.1	3024	10.1	960	19.2	3024	3.7	960	8.3	3024	5.5	960	7.9
40–49 years	2192	11.3	1112	13.7	2192	13.9	1112	20.1	2192	7.3	1112	7.9	2192	9.9	1112	10.8
50–59 years	960	21.9	840	24.2	960	23.8	840	25.5	960	12.5	840	12.9	960	10.9	840	12.4
60–69 years	128	24.5	80	26.4	128	25.8	80	30.1	128	15.5	80	17.6	128	15.8	80	17.2
Elementary	8368	9.9	3000	14.1	8368	12.3	3000	20.5	8368	5.4	3000	9.6	8368	7.5	3000	12.5
High school	920	4.3	584	9.6	920	8.7	584	12.3	920	2.6	584	4.1	920	4.4	584	5.5
Non PhA	3928	19.7	2128	20.1	3928	24.8	2128	28.9	3928	10.8	2128	12.5	3928	14.8	2128	17.8
Yes PhA	5360	3.2	1456	4.4	5360	5.5	1456	6.7	5360	2.5	1456	3.8	5360	2.8	1456	4.1
Non MD	4120	18.2	2144	19.2	4120	24.1	2144	26.8	4120	10.2	2144	11.8	4120	13.8	2144	16.5
Yes MD	5168	4.4	1440	5.1	5168	6.5	1440	8.1	5168	3.1	1440	4.9	5168	4.4	1440	5.6
Non smokers	6320	9.4	2408	13.6	6320	11.3	2408	11.8	6320	4.8	2408	5.4	6320	6.7	2408	7.5
Smokers	2968	9.7	1176	14.0	2968	12.3	1176	12.9	2968	5.7	1176	6.8	2968	7.3	1176	13.3

TyG Triglyceride glucose index. BMI Body mass index. METS-IR Metabolic score for insulin resistance. SPISE-IR Single-point insulin sensitivity estimator. SD Standard deviation. PhA Physical activity. (*) Statistical significance in all cases.

**Table 4:** Multinomial logistic regression.

Header	TyG High	TyG-BMI	METS-IR High	SPISE-IR High
	OR (95% CI)	OR (95% CI)	OR (95% CI)	OR (95% CI)
Women	1	1	1	1
Men	2.59 (2.41–2.2.78)	1.36 (1.27–1.1.46)	0.84 (0.77–0.92)	1.11 (1.07–1.15)
18–29 years	1	1	1	1
30–39 years	1.06 (1.04–1.08)	1.20 (1.15–1.25)	1.33 (1.22–1.44)	1.16 (1.12–1.20)
40–49 years	1.21 (1.16–1.26)	1.30 (1.23–1.37)	1.43 (1.31–1.55)	1.25 (1.19–1.31)
50–59 years	1.56 (1.48–1.65)	1.45 (1.37–1.54)	1.51 (1.38–1.64)	1.37 (1.28–1.47)
60–69 years	1.92 (1.66–2.19)	1.94 (1.80–2.09)	2.37 (1.95–2.79)	1.96 (1.66–2.27)
Elementary	1	1	1	1
High school	1.10 (1.07–1.14)	1.15 (1.10–1.21)	1.12 (1.10–1.15)	1.15 (1.10–1.20)
Commerce	1	1	1	1
Industry	1.23 (1.16–1.30)	1.17 (1.12–1.23)	1.37 (1.28–1.47)	1.20 (1.14–1.26)
Yes physical activity	1	1	1	1
Non physical activity	10.45 (9.25–11.66)	12.33 (11.01–13.66)	11.87 (10.27–13.48)	8.31 (7.50–9.12)
Yes Mediterranean diet	1	1	1	1
Non Mediterranean diet	4.23 (3.70–4.77)	5.29 (4.80–5.79)	5.22 (4.60–5.83)	3.64 (3.19–4.10)
Non smokers	1	1	1	1
Smokers	1.53 (1.46–1.61)	1.13 (1.09–1.17)	1.09 (1.04–1.14)	1.09 (1.05–1.13)

TyG Triglyceride glucose index. BMI Body mass index. METS-IR Metabolic score for insulin resistance. SPISE-IR Single-point insulin sensitivity estimator. OR Odss ratio.

## Data Availability

Data are not available due to ethical or privacy restrictions.
